# Changes in CT morphology can be an independent response marker for patients receiving regorafenib for colorectal liver metastases: retrospective pilot study

**DOI:** 10.1186/s12885-018-4067-5

**Published:** 2018-02-05

**Authors:** Yukinori Ozaki, Junichi Shindoh, Wataru Gonoi, Yujiro Nishioka, Chihiro Kondoh, Yuko Tanabe, Shuichiro Matoba, Hiroya Kuroyanagi, Masaji Hashimoto, Toshimi Takano

**Affiliations:** 1 Department of Medical Oncology, Toranomon Hospital, 2-2-2 Toranomon, Minato-ku, Tokyo, 105-8470 Japan; 2 Hepatobiliary-pancreatic Surgery Division, Department of Digestive Surgery, Toranomon Hospital, 2-2-2 Toranomon, Minato-ku, Tokyo, 105-8470 Japan; 3 Department of Radiology, University of Tokyo Hospital, 7-3-1 Hongou, Bunkyou-ku, Tokyo, 113-8655 Japan; 4 Colorectal Surgery Division, Department of Digestive Surgery, Toranomon Hospital, 2-2-2 Toranomon, Minato-ku, Tokyo, 105-8470 Japan; 5 Okinaka Memorial Institute for Medical Disease, 2-2-2 Toranomon, Minato-ku, Tokyo, 105-8470 Japan

**Keywords:** Colorectal liver metastases, Chemotherapy, RECIST, Morphologic response, Regorafenib

## Abstract

**Background:**

Regorafenib is a multi-kinase inhibitor, which was shown to be effective for patients with metastatic colorectal cancer refractory to standard therapies. However, its patterns of response has not yet been fully understood.

**Methods:**

Clinical records of 10 patients who received regorafenib for evaluable colorectal liver metastases were reviewed. Response to chemotherapy was evaluated with the RECIST and morphologic response criteria, and its clinical relevance was analyzed.

**Results:**

All patients received multiple lines of fluorouracil-based chemotherapy before regorafenib. The median follow-up duration after introduction of regorafenib was 4.9 months (range, 2 to 12.5 months). Median number of chemotherapy cycles was 2 (range, 1 to 15). In size-based response evaluation, 4 patients presented SD and 6 patients showed PD according to the RECIST. In non-size-based response evaluation, 3 patients were classified as optimal morphologic response and 7 patients were categorized as suboptimal morphologic response. Patients who presented optimal morphologic response showed significantly longer progression-free survival compared with those presented suboptimal response (median, 4.9 months vs. 0.7 months; *P* = 0.028), while size-based response evaluation could not well stratify patient prognosis.

**Conclusion:**

Non-size-based CT morphologic response could be a potential alternative response marker for patients treated with regorafenib.

**Electronic supplementary material:**

The online version of this article (10.1186/s12885-018-4067-5) contains supplementary material, which is available to authorized users.

## Background

Regorafenib is a small-molecule multikinase inhibitor that has been proven to be effective for prolonging the survival of patients with treatment-refractory metastatic colorectal cancer. [1, 2] However, the reported outcomes in former randomized studies were not clinically sufficient, with a median progression-free survival (PFS) period of 2.0 to 3.2 months and a median overall survival (OS) period of 6.4 to 8.8 months. Although approximately 40% to 50% of patients achieved disease control for at least 6 weeks, the objective response rates were only 1% to 4%, and most of the cases were categorized as stable disease (SD) according to the Response Evaluation Criteria in Solid Tumors (RECIST). Nevertheless, in actual clinical settings, several cases exhibiting a relatively long response to regorafenib (in terms of tumor markers) without any size response or even with an increase in the size of the tumor have been encountered. Therefore, response evaluations might require a multifaceted approach to evaluate the effects of this biologic agent accurately.

Although a size-based response evaluation according to the RECIST is the gold standard, [3] some reports suggested the morphologic change should be taken into account especially in patients treated with a regimen that includes bevacizumab. [4–10] The computed tomographic (CT) morphologic response criteria [4, 6] were proposed to assess the non-size-based response to chemotherapy (e.g., the pathologic response to chemotherapy), and the efficacy of these criteria has been validated in medical [11] and surgical [8] populations. Because regorafenib has an inhibitory effect on the same signal pathways that other anti-vascular endothelial growth factor (VEGF) antibodies block, its effect might need to be evaluated using non-size-based criteria similar to those required for bevacizumab.

The objective of this study was to clarify the presence of non-size-based responses in patients treated with regorafenib and to investigate the potential clinical impact of a morphologic response in patients treated with salvage-line therapy.

## Methods

### Study population

By searching a database of medical records at Toranomon Hospital, we identified 29 patients who had received regorafenib for metastatic colorectal cancer after standard chemotherapy including fluorouracil, oxaliplatin and irinotecan with or without molecularly targeted drugs between the period of January 2013 and April 2016. Among these, 10 patients who had liver metastases evaluable with pre- and post-chemotherapy contrast enhanced computed tomography (CECT) were retrospectively reviewed. All the analyses in the current study were performed in accordance with the ethical guidelines for clinical studies at Toranomon Hospital and were approved by the Institutional Review Board.

### Imaging analysis

The imaging analysis was performed using CECT scans, and the images were reviewed by a radiologist (WG) who was blinded to the clinical data. The response to chemotherapy was determined according to RECIST ver.1.1 [12] and the morphologic response criteria. [4] The morphologic criteria were defined as follows: group 1, homogeneous low attenuation with a thin, sharply defined tumor-liver interface; group 3, heterogeneous attenuation with a thick, poorly defined tumor-liver interface; and group 2, intermediate morphology that could not be rated as group 1 or 3 (Additional file 1: Figure S1). An optimal morphologic response was defined as a change in morphology from group 3 or 2 to group 1, and a suboptimal morphologic response was defined as a change in morphology from group 3 to group 2 or the absence of remarkable changes in morphology, as previously described. [4, 6] In patients with multiple tumors, the morphologic response was assigned based on the response seen in the majority of tumors.

### Statistical analysis

Continuous variables were compared using the Mann-Whitney *U* test, and categorical variables were compared using the chi-squared test or the Fisher exact test, where appropriate. Interobserver agreements in image reading were evaluated using the kappa value. The OS period and the PFS period were determined from the date of initial treatment with regorafenib until the date of death or the initial tumor progression, respectively. All the cases without specific prognostic events were censored at the date of the last follow-up examination. The survival curves were generated using the Kaplan-Meier method and were compared using a log-rank test. Data on serum tumor markers were collected for an exploratory analysis. The statistical analyses were performed using JMP (version 11.0; SAS Institute Inc., USA). All the statistical tests were two-sided, and significance was set at *P* < 0.05.

## Results

### Patient characteristics

The baseline demographics and clinical characteristics of the 10 patients who had evaluable liver metastases are listed in Table 1. The median age was 63 years (range, 39–83 years), and 8 patients were male (80%) and 2 patients were female (20%). The primary tumor site was the colon in 2 patients (20%) and the rectum in 8 patients (80%). Extrahepatic disease was observed in all the patients. All the patients received fluorouracil-based chemotherapy before regorafenib, and the number of prior chemotherapy regimens was 1 in 1 patient (10%), 2 in 3 patients (30%), 3 in 3 patients (30%), and 4 in 3 patients (30%). These prior chemotherapy regimens included oxaliplatin, irinotecan, bevacizumab, cetuximab, panitumumab and TAS-102. Bevacizumab was used in all the patients, and oxaliplatin was used in nine patients (90%). Eight patients (80%) were treated with irinotecan regimens. Cetuximab was used in 1 patient (10%), and panitumumab was used in 4 patients (40%). All the patients whose RAS status was wild-type received either cetuximab or panitumumab as a prior chemotherapy regimen. Three patients received TAS-102 before regorafenib. The median follow-up duration was 4.9 months from the initiation of regorafenib treatment (range, 2 to 12.5 months). The median number of treatment cycles was 2 (range, 1–15). The reasons for discontinuation were progressive disease (PD) in 3 patients and adverse events in 4 patients; 1 patient was continuing to receive treatment at the time of the analysis.

**Table 1:** Baseline demographics and clinical characteristics

	Number of patients	%
Age, years		
Median	63	
Range	39–83	
Sex		
Male	8	80
Female	2	20
Primary tumor		
Colon	2	20
Rectum	8	80
Extrahepatic disease		
Present	10	100
Absent	0	0
Number of prior chemotherapy lines		
1	1	10
2	3	30
3	3	30
4	3	30
Prior chemotherapy regimen		
Oxaliplatin	9	90
Irinotecan	8	80
Bevacizumab	10	100
Cetuximab	1	10
Panitumumab	4	40
TAS-102	3	30
RAS status		
Wild	5	50
Mutation	5	50

### Response to chemotherapy

**Fig. 1:**
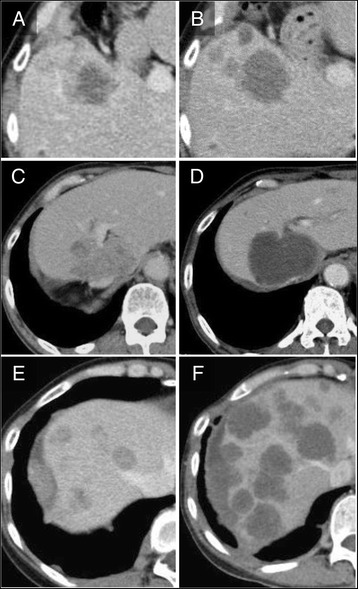
Patients presenting optimal morphologic responses during treatment with regorafenib. **A, B**. Optimal morphologic response observed at 175 days after initiation of regrafenib. Size of tumor was slightly increased (+ 6.0%) with reduction in CT density. CEA levels were 911 ng/ml (A) and 840 ng/ml (B), respectively. **C, D.** Optimal morphologic response observed at 105 days after initiation of regrafenib. Size of tumor was slightly increased (+ 16.0%) with evident change in CT texture of tumor. CEA levels were 860 ng/ml (C) and 910 ng/ml (D), respectively. **E, F.** Optimal morphologic response observed at 90 days after initiation of regrafenib. Although there was remarkable progression of tumor in number and size, serum CEA levels decreased from 91.7 ng/ml (E) to 66.1 ng/ml (F)

**Fig. 2:**
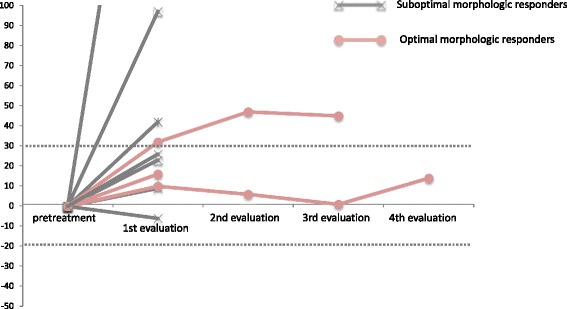
Changes in tumor size according to morphologic response

Among the 10 patients, optimal morphologic responses were observed in 3 patients, and 7 patients did not show any changes in CT morphology during the treatment. According to the RECIST, the response rate was 0%: 4 patients had SD, and 6 patients had PD. In all 3 patients who achieved an optimal morphologic responses, the size of the tumor nodule had increased at the time of the initial diagnosis of an optimal morphologic response (+ 6.0% [SD], + 16.0% [SD], and + 31.5% [PD], respectively) (Figs. 1, 2). Although these patients had histories of bevacizumab as a first- or second-line treatment, no morphologic changes were seen at the time of the former treatment with bevacizumab.

Serum tumor markers, such as carcinoembryonic antigen (CEA), were analyzed in all the patients at the time of radiographic evaluation during regorafenib treatment. The median rate of CEA increase was 2.02 (range, 0.75–32.3) in 7 patients with a suboptimal morphologic response, whereas it was 0.95 (range, 0.72–1.59) in 3 patients with an optimal morphologic response. The median duration of the morphologic response was 3.4 months, and there were no significant increases in the CEA levels during the period of the morphologic responses in all 3 patients.

### Prognostic outcomes

**Fig. 3:**
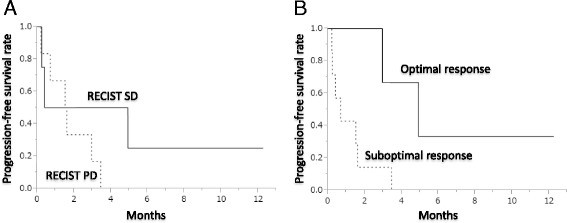
Progression-free survival stratified by the RECIST and CT morphologic response. **A** Progression-free survival stratified by the RECIST; **B**. Progression-free survival stratified by the CT morphologic response

In the entire cohort, the median PFS was estimated as 1.6 months and the median OS was 4.9 months after the initiation of regorafenib. When stratified according to the RECIST, a difference in PFS between the SD and PD patients was not evident (median PFS, 2.7 months vs. 1.6 months; *P* = 0.30). However, when compared according to whether or not a morphologic response was observed, patients with optimal morphologic responses showed a significantly longer PFS than those with suboptimal responses (median PFS, 4.9 months vs. 0.7 month; *P* = 0.028) (Fig. 3). Similar analyses for OS showed no prognostic differences between the patients with an SD according to the RECIST and those with PD (median OS, 4.7 months vs. 4.9 months; *P* = 0.99), while those with optimal morphologic responses tended to survive longer than those with suboptimal responses (median OS, not reached vs. 4.6 months; *P* = 0.15). Univariate Cox-regression confirmed that PFS or OS was not correlated with the other potential prognostic factors including age, performance status, number of chemotherapy lines, history of treatment with FOLFOX or FOLFIRI, history of TAS102 administration, presence of toxicity, original tumor stage, or original tumor location.

### Difference in background characteristics between the optimal response group and the suboptimal response group

Table 2 compares the background characteristics between the patients with an optimal response and those with a suboptimal response. Although a statistical comparison is difficult because of the limited number of cases, the group with an optimal morphologic response tended to be younger and had completed a larger number of treatment cycles without discontinuation because of toxicity before disease progression.

**Table 2:** Comparison of background characteristics between the patients with optimal response and suboptimal response

	Optimal response	Suboptimal response
Age, years		
Median	57	71
Range	36–67	55–83
Gender		
Male	1	1
Female	2	6
Primary (colon/rectum)		
	0	2
	3	5
RAS (wild/mutant)		
wild	2	3
mutant	1	4
Performance status		
0	0	2
1	3	4
2	0	1
No. of chemotherapy lines		
2	0	1
3	0	3
4	2	1
5	1	2
Cycles of regorafenib		
Median	4	1
Range	1–15	1–3
Reasons for discontinuation		
Disease progression	2	3
Toxicity	0	4
Best response in RECIST		
SD	2	2
PD	1	5
Best change in size		
Median	+ 16.0%	+ 25.5%
Range	+ 1.4% to + 31.5%	−6.2% to + 280.6%

*Abbreviations. SD* stable disease, *PD* progressive disease

## Discussion

In this study, we examined the existence of a non-size-based morphologic response in patients receiving regorafenib for colorectal liver metastases that were refractory to standard chemotherapies. An optimal morphologic response was correlated with a significantly longer PFS, and patients who exhibited an optimal morphologic response seemed to achieve better disease control.

The CT morphologic response to chemotherapy has been reported to be a good prognostic indicator in both medical [4, 11] and surgical [4, 6, 8] populations with colorectal liver metastases, especially in patients treated with bevacizumab. The reported strength of a CT morphologic response is that it may predict differences in pathological response “quality,” [7, 13, 14] and a strong correlation between CT morphology and the pathological viability of tumors has been confirmed in previous studies. [4, 6, 8] The potential utility of the CT morphologic response for medical populations with unresectable tumors is that it could be used in decision-making processes to reduce the intensity of treatment during the maintenance phase of intensive chemotherapy. However, its usefulness in salvage-line treatment has never been reported.

The current study was conducted to clarify the existence of a non-size-based response among patients receiving regorafenib and to investigate its clinical relevance. Three out of 10 patients showed evident changes in CT texture in liver metastases presenting with relatively stable serum CEA levels (Fig. 1). Interestingly, a significantly longer PFS was observed in patients with optimal morphologic responses, though all 3 patients exhibited an increase in tumor size at the time of the diagnosis of the morphologic response. Although previous studies have confirmed that the morphologic response occurs independently of the size response, [4, 6, 8] given that 40%–60% of patients with the optimal responses also exhibited size-based responses, [6, 8] the patterns of response observed in the present study seem to differ from those reported for first-line chemotherapy.

The actual clinical impact of a CT morphologic response in salvage-line therapy remains uncertain. However, the current results are potentially encouraging in terms of a multifaceted approach for evaluating whether regorafenib is actually effective. Given that most patients showed disease progression and suffered from toxicity as early as the initial evaluation, the early stratification of patients who might truly benefit from regorafenib therapy could contribute to the improved supportive management of patients with advanced-stage colorectal cancer.

The limitations of the current study include its retrospective nature, the strictly selected population, and the limited number of cases. However, this is the first report to describe the presence of a non-size-based response to regorafenib, and to suggest the prognostic advantage of an optimal morphologic response. A validation study using a large number of patients is strongly recommended to confirm the present results and to improve the management of patients receiving salvage-line chemotherapy with regorafenib.

## Conclusions

In conclusion, a non-size-based CT morphologic response could be a potential prognostic marker for patients receiving regorafenib for unresectable colorectal liver metastases. A multifaceted evaluation might be required to evaluate the response to regorafenib accurately.

## Additional file

Additional file 1: Figure S1. CT morphologic criteria and tumor thickness at the tumor-liver interface. (with permission of Springer) [8] **a** Group 1 CT morphology. **b** Group 2 CT morphology. **c.** Group 3 CT morphology. **d** Typical tumor thickness at the tumor-liver interface in group 1 morphology (arrows). **e** Tumor-liver interface in group 2 morphology (double-ended arrow). **f** Thick tumor-liver interface in group 3 morphology (double-ended arrow). (PPTX 1708 kb)

## Abbreviations

CEA: carcinoembryonic antigen
CECT: chemotherapy contrast enhanced computed tomography
CT: computed tomography
OS: overall survival
PD: progressive disease
PFS: progression-free survival
RECIST: Response Evaluation Criteria in Solid Tumors
SD: stable disease
VEGF: vascular endothelial growth factor
