# Supplementary figures and images for: Changes in CT morphology can be an independent response marker for patients receiving regorafenib for colorectal liver metastases: retrospective pilot study
Source: BMC Cancer. 2018 Feb 5;18:138. doi: 10.1186/s12885-018-4067-5 (PMC5800281; doi:10.1186/s12885-018-4067-5)

## Slide 1
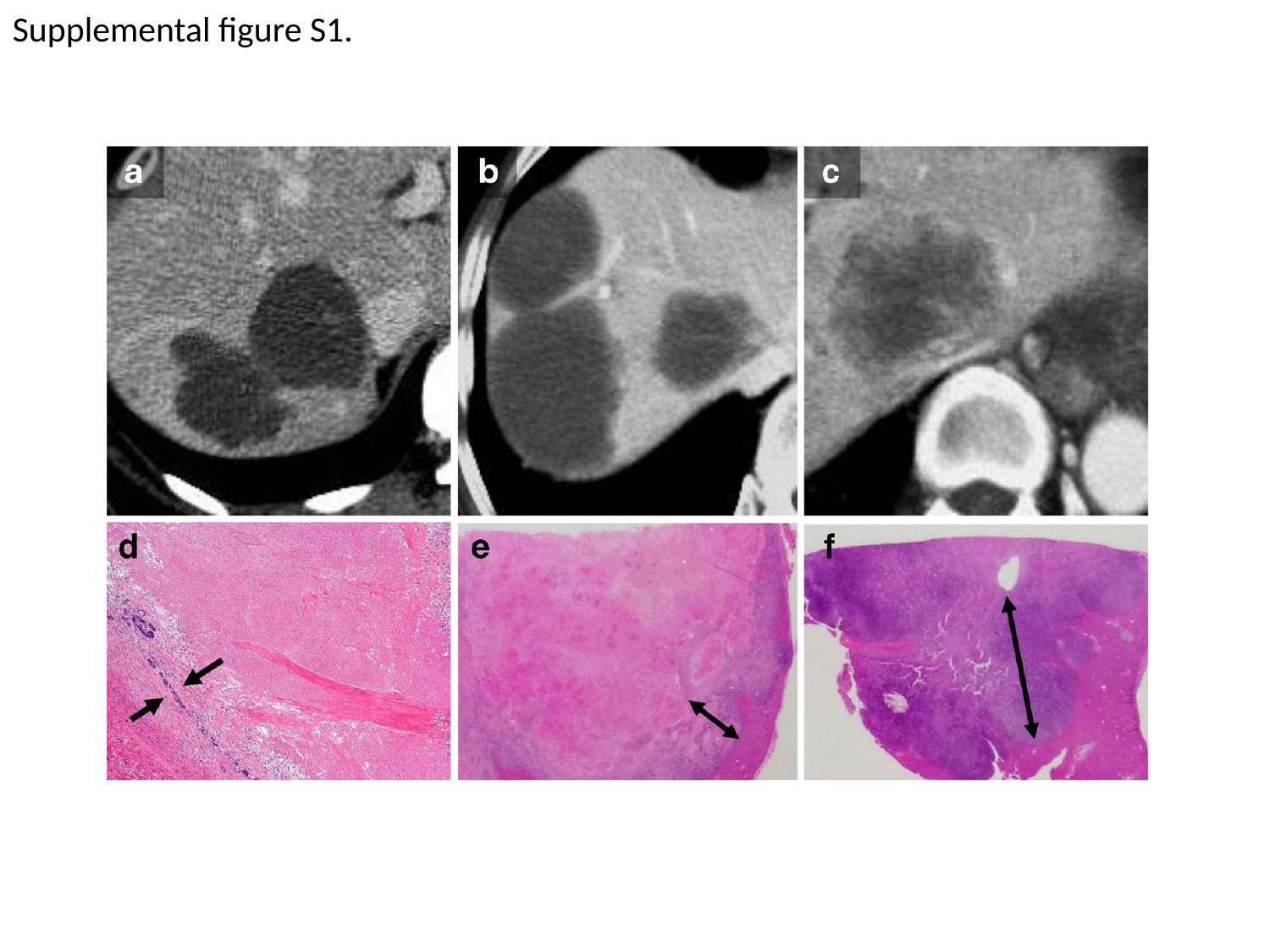

Supplemental figure S1.

Supplement: Additional file 1: Figure S1. — CT morphologic criteria and tumor thickness at the tumor-liver interface. (with permission of Springer) [8] a Group 1 CT morphology. b Group 2 CT morphology. c. Group 3 CT morphology. d Typical tumor thickness at the tumor-liver interface in group 1 morphology (arrows). e Tumor-liver interface in group 2 morphology (double-ended arrow). f Thick tumor-liver interface in group 3 morphology (double-ended arrow). (PPTX 1708 kb) [file 12885_2018_4067_MOESM1_ESM.pptx]
